# Genome Wide Host Gene Expression Analysis in Chicken Lungs Infected with Avian Influenza Viruses

**DOI:** 10.1371/journal.pone.0153671

**Published:** 2016-04-12

**Authors:** Pradip B. Ranaware, Anamika Mishra, Periyasamy Vijayakumar, Pradeep N. Gandhale, Himanshu Kumar, Diwakar D. Kulkarni, Ashwin Ashok Raut

**Affiliations:** 1 Pathogenomics Laboratory, ICAR -National Institute of High Security Animal Diseases, OIE Reference lab for Avian Influenza, Bhopal, Madhya Pradesh, India; 2 Laboratory of Immunology, Department of Biological Sciences, Indian Institute of Science Education and Research, Bhopal, Madhya Pradesh, India; University of Hyderabad, INDIA

## Abstract

The molecular pathogenesis of avian influenza infection varies greatly with individual bird species and virus strain. The molecular pathogenesis of the highly pathogenic avian influenza virus (HPAIV) or the low pathogenic avian influenza virus (LPAIV) infection in avian species remains poorly understood. Thus, global immune response of chickens infected with HPAI H5N1 (A/duck/India/02CA10/2011) and LPAI H9N2 (A/duck/India/249800/2010) viruses was studied using microarray to identify crucial host genetic components responsive to these infection. HPAI H5N1 virus induced excessive expression of type I IFNs (IFNA and IFNG), cytokines (IL1B, IL18, IL22, IL13, and IL12B), chemokines (CCL4, CCL19, CCL10, and CX3CL1) and IFN stimulated genes (OASL, MX1, RSAD2, IFITM5, IFIT5, GBP 1, and EIF2AK) in lung tissues. This dysregulation of host innate immune genes may be the critical determinant of the severity and the outcome of the influenza infection in chickens. In contrast, the expression levels of most of these genes was not induced in the lungs of LPAI H9N2 virus infected chickens. This study indicated the relationship between host immune genes and their roles in pathogenesis of HPAIV infection in chickens.

## Introduction

Avian influenza virus (AIV) is classified based on their virulence in chickens into low pathogenic avian influenza virus (LPAIV) and highly pathogenic avian influenza virus (HPAIV) [1]. Pathogenicity differences observed between HPAIV and LPAIV might be related to specific characteristics of virus strains, tissue tropism and host responses [2]. Low pathogenic avian influenza virus usually produces subclinical infections in chickens [3]. In contrast, highly pathogenic avian influenza infections lead to rapid onset of severe, contagious systemic disease with 100% mortality within 48 hours of infection [3].

LPAIV mainly replicate in the respiratory and the digestive tract of chicken. However, Post et al reported the presence of H7N1 LPAIV in organs beyond the respiratory and the gastrointestinal tract indicating that the virus could spread systemically after an intranasal/intratracheal infection [2]. HPAIV have a polybasic hemagglutinin (HA) cleavage site sensitive to ubiquitous proteases which enables them to replicate in multiple organs, thereby causing severe systemic disease with high mortality in gallinaceous poultry [3,4]. Although cleavage activation of HA is necessary for the pathogenicity of HPAIV, host factors involved in the molecular pathogenesis of HPAIV infection in chickens have not yet been identified [5].

H9N2 virus is among the most commonly seen influenza viruses in domestic poultry populations [6] and it is unique among LPAIV in that they infect a wide variety of species including various domestic poultry species, pigs, and humans [7, 8]. Because H9N2 viruses infect a number of species, crossing the species barriers, it holds potential to impact as emerging virus [8, 9]. Hence, it warrants further studies on the molecular pathogenesis of H9N2 viruses in both humans and avian species [6].

Dysregulation of the innate immune response may be a critical determinant of the severity and the outcome of the influenza infection [10, 11]. Dysregulation of cytokines and chemokines in lungs is reported to cause tissue damage and high mortality in mice [12, 13], ferrets [14], macaques [15, 16] and in *in vitro* infection models [17]. Little is known about dysregulation of cytokines and chemokines in chickens after HPAIV or LPAIV infection [18, 19]. Previous studies do not support the hypothesis that dysregulation of proinflammatory cytokines and chemokines are the critical determinant for the severity and the outcome of the influenza infection in chickens infected with HPAIVs [18, 20]. Only few studies have compared the immune response in chickens to LPAIV and HPAIV infection [2, 5, 18, 20] and their focus was on a very limited number of genes using RT qPCR. As a result, these studies do not give a global overview of interactions between the host (chickens) and the different pathotypes of influenza virus. Most of the current host-pathogen interaction research is inclined towards human model systems [21] and the knowledge of the host-pathogen interaction and the molecular mechanisms underlying the pathogenesis of HPAIV or LPAIV infection in avian species is still merger. Hence, there is a need to understand the virus–host interactions in avian species, because the wild and domestic birds are act as the reservoirs for most of the influenza A viruses [22].

In this study, we show that HPAI H5N1 virus infection in chickens intensely triggers the genes related to inflammatory and innate immune responses. However, this intense host antiviral response is unsuccessful in controlling the rapidly progressing infection and results in high mortality in chickens. In contrast, the expression levels of most of these genes remain unchanged in the lung tissue of chickens infected with LPAI H9N2 virus. Our microarray dataset provides insight into the potential molecular pathogenesis and also reveals the molecular basis of differential response of chicken to different pathotypes of AIV.

## Materials and Methods

### Ethics statement

The animal experiments were carried out at Biosafety level 3+ containment facility of ICAR- National Institute of High Security Animal Diseases (ICAR-NIHSAD), Bhopal, India. The experiments were approved by the institutional animal ethics committee of ICAR-NIHSAD (Approval no. 68/IAEC/HSADL/12 dated 11.05.2012), and performed under the guidance of the Committee for the Purpose of Control and Supervision of Experiments on Animals (CPCSEA), Ministry of Environment and Forests, Govt. of India.

### Viruses

Avian influenza LPAI H9N2 virus (A/duck/India/249800/2010) and HPAI H5N1 virus (A/duck/India/02CA10/2011) were obtained from avian Influenza virus repository of ICAR-NIHSAD, Bhopal, India. The stock virus was prepared by propagating and titrating the viruses in the allantoic cavities of 12-day-old embryonated duck eggs.

### Animal experimental design

A total of nine, six weeks old, specific pathogen–free chicken were divided into three groups of three birds each. Chicken of group 1 were intranasally inoculated with 10^6^ EID_50_ of the HPAI H5N1 virus (A/duck/India/02CA10/2011). Group 2 was inoculated with 10^6^ EID_50_ of the LPAI H9N2 virus (A/duck/India/249800/2010). Chicken in control group were inoculated with phosphate buffer saline (PBS). The birds were observed daily for clinical signs. The clinical symptoms like dullness, lacrimation, cyanotic combs and wattles gradually appeared and were full blown by 2 days post infection (dpi) in HPAI H5N1 infected chicken. Mild clinical symptoms like depression, decreased feed and water consumption and ruffled feathers indicating sickness were observed in LPAI H9N2 virus infected chicken at 6 dpi. The birds from HPAI H5N1 virus infected group were sacrificed at 2 (dpi) and the birds from LPAI H9N2 virus infected group and control group were sacrificed at 6 dpi. None of the birds inoculated with either virus died before the end points of experiments. All birds were sacrificed by cervical dislocation. Lung tissues were collected in RNA later from all sacrificed birds and stored at -80°C. The virus infection in lungs was confirmed by tissue inoculation in embryonated chicken eggs and hemagglutination (HA) assay.

### Total RNA isolation and microarray hybridization

Total RNA was isolated from 2 lung tissue samples from control group and 3 lung tissue samples each from group 1 and group 2 using TRIzol reagent (Invitrogen, USA) with the Qiagen’s RNeasy minikit (Qiagen, Germany). The integrity of RNA was checked using the Bioanalyzer. The Custom Chicken 8x60k array on Agilent platform (AMADID G4102A_059389) was synthesized from Genotypic Technology Pvt. Ltd by printing the sets of oligonucleotide. Apart from the 43603 probes present in the commercially available chicken oligio Micryoarray AMADIA: 026441 (4x 44K), this custom array (8x60k) had 14256 additional probes. These additional probes included ESTs of brain, liver, and lung tissues and the genes listed in Influenza A pathway in Kyoto Encyclopedia of Genes and Genomes (KEGG). The customization was done to enhance the depth of expression profiling. Total RNA was labeled using Agilent Quick Amp labeling kit by standard procedure. cRNA was purified using Qiagen RNeasy column. Concentration and amount of dye incorporated into labeled cRNA was determined using Nanodrop ND-1000 UV-VIS spectrophotometer. Samples that passed the QC for specific activity were taken for hybridization. 600 ng of labeled cRNA were hybridized on the specific arrays using the Gene Expression Hybridization kit in Sure Hybridization champers at 65°C for 16 hrs. Hybridized slides were washed using Agilent Gene Expression wash buffers. Washed microarray slides were scanned on a GS600D scanner (Agilent Technologies).

### Microarray data analysis

Data extraction from images was done using Feature Extraction software version 10.7. Percentile shift normalization method was used for normalization, where the locations of all the spot intensities in an array were adjusted. The normalized raw data was then statistical analyzed using GeneSpring GX 12.5 software (Agilent) to identify differentially expressed genes. The microarray data have been deposited in NCBI Gene Expression Omnibus (Accession number GSE65231). The normalized raw data results obtained with HPAI- or LPAI-infected lungs were compared to those obtained with control. We compared both the pathotypes with the control, in order to study the molecular basis of response to infection with these individual virus pathotypes. Gene expression ratios compared with the reference control were calculated and log2 transformed. All the fold change expression values represented in the manuscript are provided as log base 2. The p-value ≤ 0.05 and fold change ≥1 (log2 transformed value) was used to determine significant differential expression. Functional classification of the genes was performed for gene ontology (GO) in Database for Annotation, Visualization and Integrated Discovery (DAVID) and pathway analysis in Kyoto Encyclopedia of Genes and Genomes (KEGG). The GO terms of immune related biological process and molecular function with p value <0.05 are discussed. The KEGG pathways involved in immune related function and viral pathogenesis were highlighted for understanding the host response to HPAI H5N1 and LPAI H9N2 infection in chicken.

### RT qPCR Assays

The differential expression data was validated by one step RT qPCR. Total RNA was isolate using TRIzol ® Reagent (Invitrogen, USA) with the Qiagen’s RNeasy minikit (Qiagen, Germany) from the same lung tissue samples used for the microarray analysis. Primers used for RT qPCR were as follows: β-actin, IFNA, IFNG, IFNAR2, STAT3, IRF1, and IRF10 [23], ILIB [6], TLR3, and TLR-15 [24], and Mx1 [25]. RT qPCR was done on StepOnePlus Real-Time PCR System (Applied Biosystems) using SYBR Green chemistry. The following amplification conditions were used: 2 min at 50°C, 10 min at 95°C, followed by 40 amplification cycles (15 sec at 95°C and 1 min at 60°C). The data obtained from the RT-qPCR was analysed by Schmittegen and Livak method [26]. The data was normalized using β-actin as the internal control gene. The ΔΔCt value was calculated by difference in normalized Ct value (ΔCt) from infected samples to the ΔCt from non-infected samples. The ΔΔCt value is transformed into 2^-ΔΔCt^ value as the estimated gene expression fold change value.

## Results

### Host gene response to HPAI H5N1 virus infection

**Fig 1:**
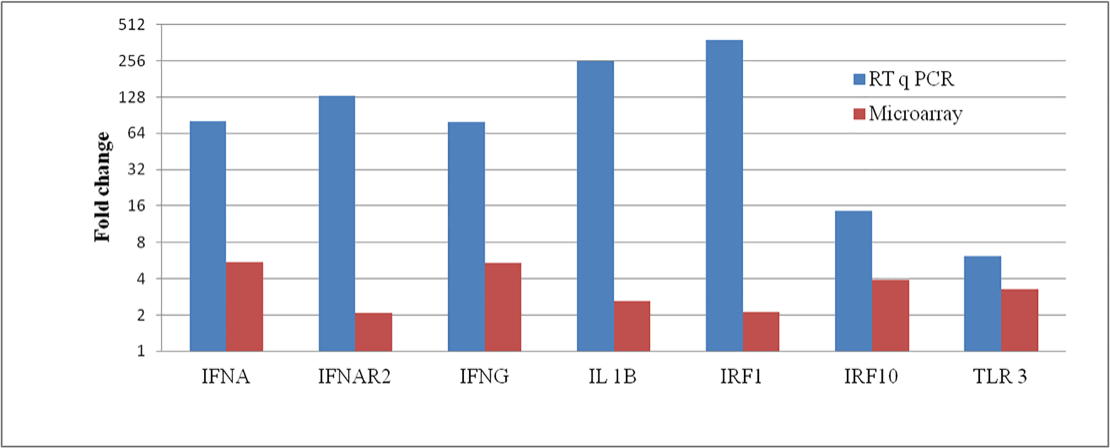
Validation of microarray data by RT qPCR. Relative amount of differentially expressed genes in HPAIV H5N1 infected as compared to non infected lung tissues using RT qPCR.

Agilent chicken microarray chips were used to identify genes contributing to the innate immune response to HPAI H5N1 influenza virus infected lung tissues. Hemagglutinin titer of the HPAI H5N1 virus (A/duck/India/02CA10/2011) in infected lung tissues was 2^8^. As compared to the control, approximately 5550 genes were significantly differentially expressed at the cut-off of p-value ≤ 0.05 and fold change ≥1 (log2 transformed value) in HPAIV infected lungs tissues. Of these, 4785 genes were upregulated and 765 genes were down regulated (Table 1). We identified several immune genes upregulated including TLR3, TLR6, TLR15, IFIH1/MDA, NLRC5, MX1, IFITM5, OAS, TRIM25, SOCS3, STAT1, IL1B, IL18, IL13, IFNA, IFNG etc. and downregulated including IL12RB2, TRIM13, TGFB3, ADAT2 etc. in the lung tissue of chickens infected with HPAI H5N1 virus. The results of the microarray data were supported using RT qPCR (Fig 1). The gene expression fold-change values in microarray data were not identical to RT qPCR data; however the overall trend in gene expression changes was consistent in both dataset. Similar kinds of discrepancy in gene expression fold change values were reported previously [27, 28]. This general discrepancy in gene expression fold change values in microarray and RT qPCR techniques has been the subject of previous research [29, 30].

**Table 1:** Differentially expressed genes in response to infection with HPAIV H5N1 and LPAIV H9N2 virus.

Infection condition	Differentially expressed genes (+/- ≥1folds, p < 0.05)	Up-regulated genes	Down-regulated genes
HPAIV H5N1	5550	4785	765
LPAIV H9N2	2992	2552	440

**Fig 2:**
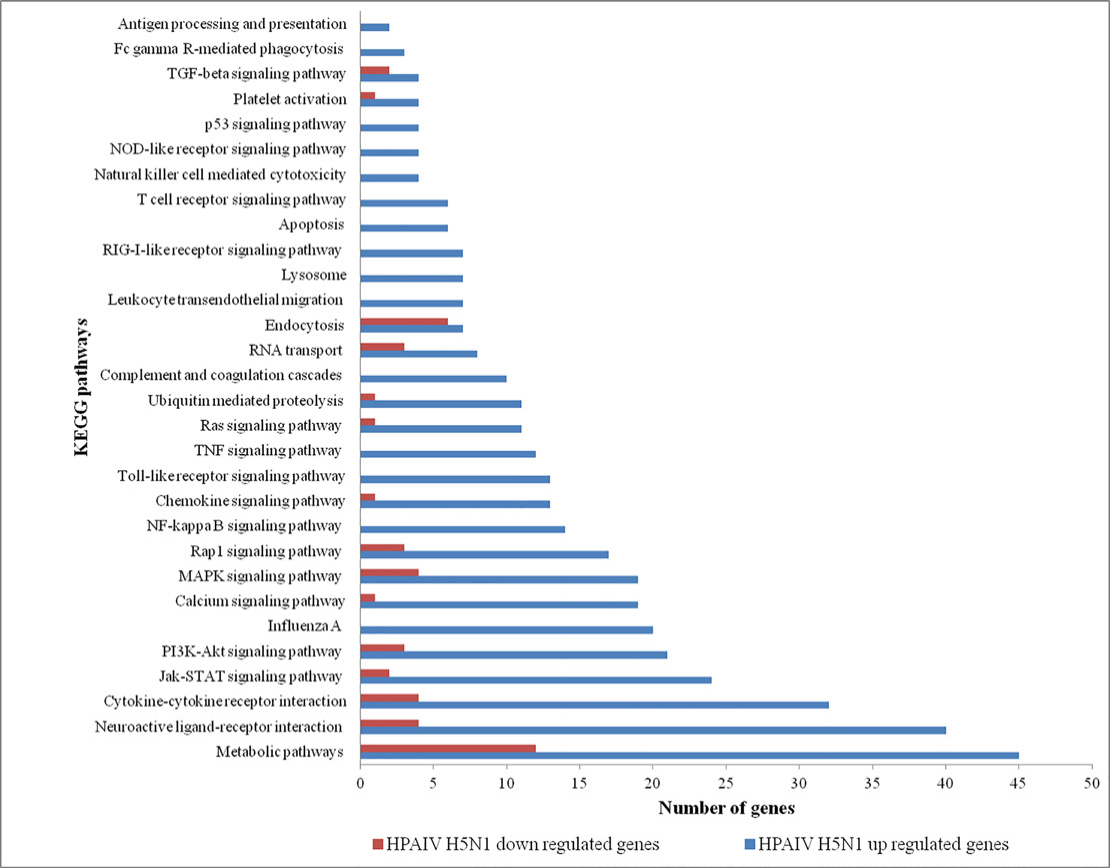
KEGG pathways enriched in response to HPAIV H5N1 infection. Up regulated and down regulated genes differentially enriched in various pathways are shown.

Gene ontology analysis of the upregulated genes revealed that the genes were involved in response to cytokine stimulus, inflammatory response, innate immunity, response to virus, regulation of adaptive immune response, and apoptosis (Table 2). Gene ontology analysis of downregulated genes revealed that the genes were involved in DNA repair, cellular response to stress, negative regulation of cellular biosynthetic process, cell cycle, positive regulation of signal transduction and transcription activator activity (Table 2). KEGG pathway analysis of HPAI H5N1 upregulated genes revealed enrichment of Influenza A pathway, Jak-STAT signaling pathway, NOD-like receptor signaling pathway, p53 signaling pathway, RIG-I-like receptor signaling pathway, Toll-like receptor signaling pathway, TNF signaling pathway etc. (Fig 2). HPAI H5N1 downregulated genes enriched the pathways such as Cytokine-cytokine receptor interaction, Jak-STAT signaling pathway, MAPK signaling pathway, PI3K-Akt signaling pathway etc. (Fig 2). However, the number of genes involved in particular pathway was quantitatively different between these up or down regulated genes list.

**Table 2:** Significantly enriched Gene Ontology terms in response to HPAIV H5N1 infection.

GO Terms	Gene Count	p-value
**Upregulated genes**		
Positive regulation of immune system process	42	1.10E-05
Positive regulation of lymphocyte activation	23	1.80E-05
Cytokine receptor activity	14	7.00E-04
Response to virus	21	8.60E-04
Inflammatory response	45	1.60E-03
Innate immunity	15	2.10E-03
Regulation of protein kinase cascade	36	2.40E-03
Negative regulation of biosynthetic process	69	3.60E-03
Regulation of interleukin-2 production	8	1.20E-02
Regulation of apoptosis	88	1.30E-02
Positive regulation of chemokine biosynthetic process	4	1.70E-02
Antigen processing and presentation of endogenous antigen	4	2.50E-02
Positive regulation of signal transduction	41	2.40E-03
Response to wounding	78	2.50E-06
Transcription factor activity	143	2.60E-10
Positive regulation of gene expression	88	1.40E-07
Regulation of interleukin-6 biosynthetic process	5	2.70E-02
**Downregulated genes**		
Response to DNA damage stimulus	15	1.50E-03
Cellular response to stress	18	5.10E-03
Negative regulation of cellular biosynthetic process	16	2.20E-02
Cell cycle	19	4.40E-02
Transcription activator activity	12	4.70E-02
Negative regulation of nitrogen compound metabolic process	14	4.90E-02

### Host gene response to LPAI H9N2 virus infection

In order to determine the genes expressed in lung tissue in response to LPAI H9N2 virus, the infected tissues were compared to control tissues. Hemagglutinin titer of the LPAI H9N2 virus (A/duck/India/249800/2010) in infected lung tissues was 2^8^. In LPAIV infected lungs tissues, approximately 2992 genes were significantly differentially expressed at the cut-off of p-value ≤ 0.05 and fold change ≥1 (log2 transformed value) as against the control tissue. Of these, 2552 genes were upregulated and 440 genes were downregulated (Table 1). The immune genes upregulated in infected tissue include IFNA3, TRIM65, TRIM71, TGFA, IL13, IL12B, IL9A, IL23A, and other housekeeping genes. In LPAIV infected tissue IFITM5, TRIM50, TGFB3, PRLR, OXSR1 etc. genes were downregulated. The RT qPCR result shows moderate up regulation of IFNA, IFNG, IFNAR2, STAT3, Mx1, ILIB, TLR3, IRF1, and IRF10 genes during LPAIV infection in chicken (**S1 Fig**).

**Fig 3:**
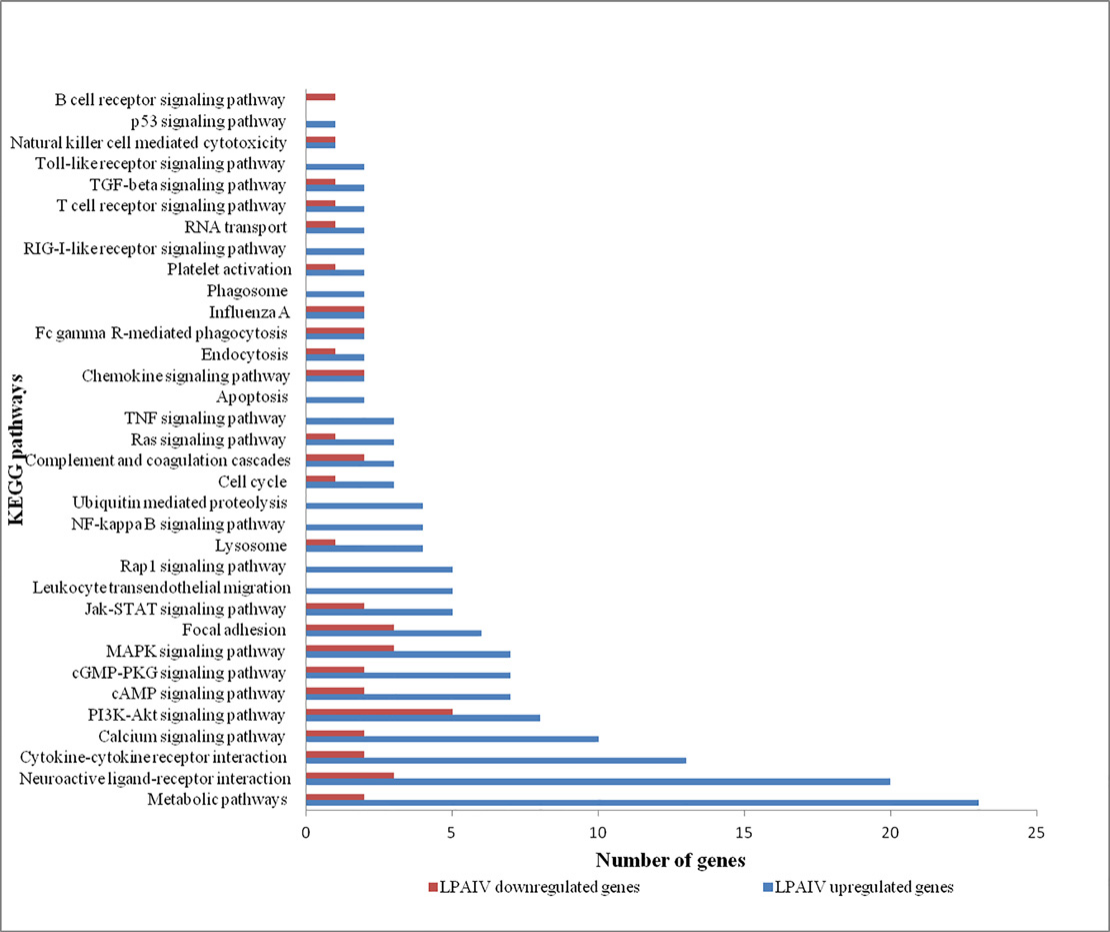
KEGG pathways enriched in response to LPAIV H9N2 infection. Up regulated and down regulated genes differentially enriched in various pathways are shown.

The following gene ontology were enriched in upregulated genes including negative regulation of transcription, cell-cell signaling, response to wounding, T cell differentiation, positive regulation of lymphocyte activation, fatty acid metabolic process and response to carbohydrate stimulus (Table 3). The GO term enriched by the downregulated genes included cellular homeostasis, calcium ion homeostasis, apoptosis and microtubule-based movement (Table 3). The genes expressed in cellular homeostasis included ATP2A2, TXN2, RHOT2, PLN, DMD, APP, and Bak1. The genes upregulated in LPAIV H9N2 infected tissues are components of the Influenza A pathway, Jak-STAT signaling pathway, MAPK signaling pathway, NF-kappa B signaling pathway, RIG-I-like receptor signaling pathway, TNF signaling pathway and Toll-like receptor signaling pathway (Fig 3). The downregulated genes are components of the B cell receptor signaling pathway, Cytokine-cytokine receptor interaction, Fc gamma R-mediated phagocytosis, Jak-STAT signaling pathway, and MAPK signaling pathway (Fig 3).

**Table 3:** Significantly enriched Gene Ontology terms in response to LPAIV H9N2 infection.

GO Terms	Gene Count	p-value
**Upregulated genes**		
Negative regulation of RNA metabolic process	30	2.10E-04
Negative regulation of transcription	35	2.70E-04
Cell -cell signaling	41	6.90E-04
Negative regulation of macromolecule biosynthetic process	38	8.40E-04
Response to wounding	36	1.70E-03
Response to abiotic stimulus	27	2.70E-03
Positive regulation of homeostatic process	7	4.30E-03
Regulation of cAMP metabolic process	10	1.90E-02
Positive regulation of lymphocyte activation	9	3.60E-02
T cell differentiation	7	4.10E-02
Cell -cell adhesion	18	4.40E-02
Regulation of nucleotide metabolic process	10	3.80E-02
Fatty acid metabolic process	16	1.10E-02
Response to carbohydrate stimulus	7	2.90E-02
**Downregulated genes**		
cellular homeostasis	7	2.30E-03
Apoptosis	5	2.00E-02
Cytoskeleton -dependent intracellular transport	3	1.20E-02
Microtubule -based movement	4	1.70E-02
Calcium ion homeostasis	4	5.00E-03

## Discussion

In this study, we examined the host responses to a highly-pathogenic H5N1 virus and a low pathogenic H9N2 virus infection in chickens. The differences in viral pathogenicity between HPAIV and LPAIV infection were related to the difference in host immune responses to these viruses.

### Immune response to HPAI H5N1 virus

The influenza viral pathogen-associated molecular patterns (PAMPs) is recognized by host pattern recognition receptors (PRRs) such as Toll-like receptors TLR3 [31] and TLR7 [32], retinoic acid-inducible gene I (RIG-I) [33] and the NOD-like receptor family member NOD-, LRR- and pyrin domain-containing 3 (NLRP3/NLRC5) [34], and activation of these PRRs induces secretion of type I interferons (IFNs), pro-inflammatory cytokines and chemokines [35]. In HPAIV infected chicken, MDA5 (IFIH1), TLR3, and NLRC5 genes were upregulated in lung tissues with fold change value of 3.7, 3.3, and 2.9 respectively.

TLRs triggers the activation of transcription factors [nuclear factor-κB (NF-κB) or IFN-regulatory factor 7 (IRF7)] responsible for the expression of type I IFNs and proinflammatory cytokines [36, 37]. In this study, TLR15 (fold change value 2.7) and TLR6 (fold change value 2.2) genes were found to be upregulated in HPAIV infected tissues. Further, MYD88 and IRF7 genes were upregulated and NF-κB signaling pathway was enriched (Fig 2). The up regulation of IFNA and IFNAR2 genes showed fold change value of 5.5 and 2.0 respectively in HPAIV infected tissues and may have been stimulated by NF-κB signaling pathway.

In the lack of RIG-I gene in chicken, MDA5 (IFIH1) gene functionally compensate the immune functions of RIG-I [38, 39]. Chicken MDA5 receptor, which uses the same signaling pathway as RIG-I, results in induction of type I IFNs and involves chicken LGP2, MAVS and IRF3/IRF7 genes [25, 40, 41]. In HPAIV infected tissues IFIH1 and IRF7 genes were upregulated indicating the activation of the type I IFN (IFNA) response in lungs. Other IFN genes namely IFNG and IFNL as well as interferon regulatory factor namely IRF1, IRF10, IRF2 and IRF8 were also upregulated in HPAIV infected tissues. Increased expressions of these genes were confirmed by RT qPCR (Fig 1). Significant increases in IFNA, IFNG and TLR3 mRNA expression are reported during HPAI infection of chicken dendritic cells [42]. The type I and III IFNs expressed through different PRRs bind to the IFNAR1/IFNAR2 and IFNGR1 and IFNGR2 receptors respectively. This receptor complex activate Jak-STAT signaling through a trimeric transcription factor complex (ISGF3) results in induction of range of IFN stimulated genes (ISGs) [43].

**Fig 4:**
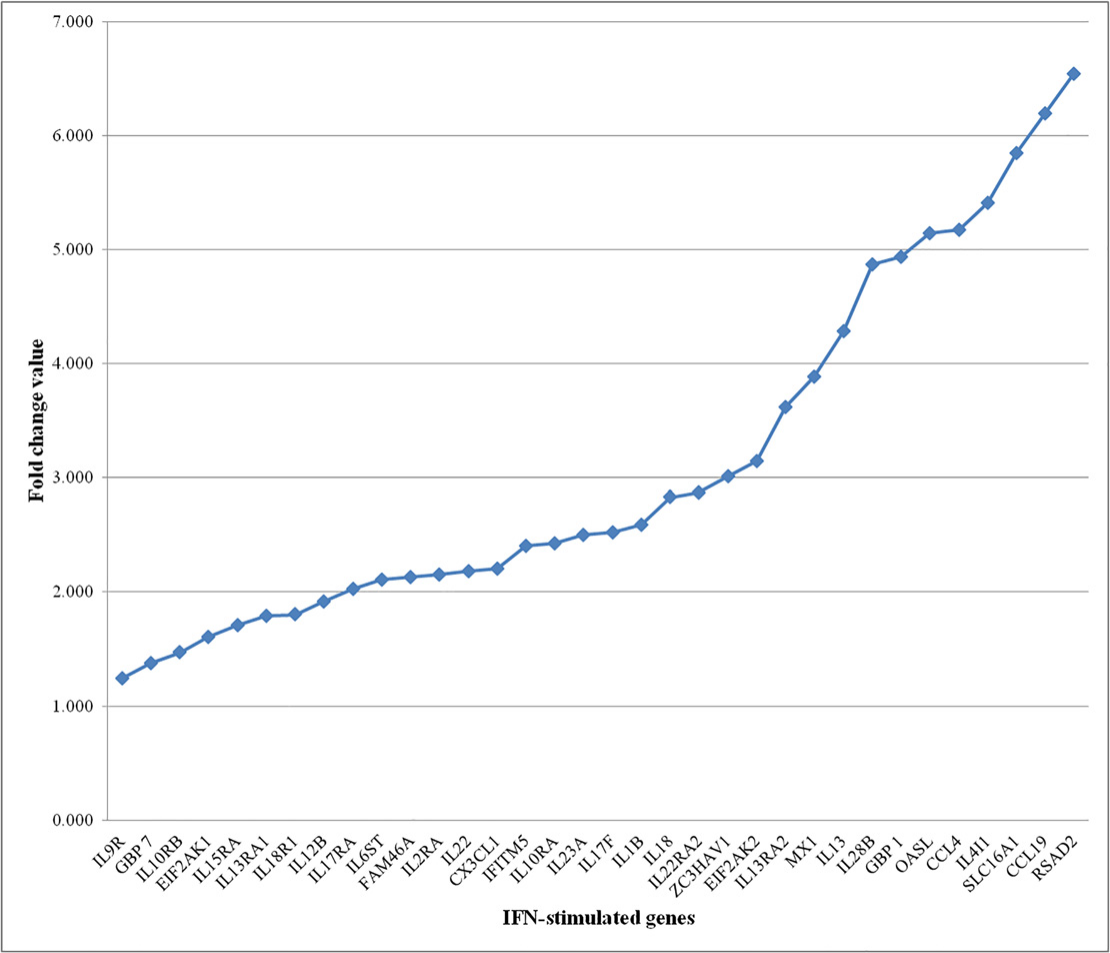
IFN-stimulated genes were expressed in response to HPAIV H5N1 infection in chicken lung tissues. The IFN-stimulated genes, pro-inflammatory cytokines and chemokines were all upregulated in HPAIV H5N1 infected lung tissues of chicken.

Amongst the IFN-stimulated genes, we found more genes were upregulated in response to H5N1 infection including the MX1, OASL, IFITM5 and IFITM3, IFIT5, EIF2AK1 (PKR) and EIF2AK2, GBP 1 and 7, SLC16A1, FAM46A, RSAD2, and ZC3HAV1 (Fig 4). Similarly, we found that a number of IFN-stimulated pro-inflammatory cytokines (IL1B, IL10RA, IL10RB, IL12B, IL13, IL13RA1, IL13RA2, IL15RA, IL17F, IL17RA, IL18, IL18R1, IL22, IL22RA2, IL23A, IL28B, IL2RA, IL4I1, IL6ST and IL9R) and the chemokines (CCL4, CCL19, CCL10, CXCL12 and CX3CL) were also upregulated in HPAIV infected lung tissues of chicken (Fig 4). The RT qPCR analysis confirmed increased ILIB, IRF1 and MX gene expression in the HPAI virus-infected lung tissues (Fig 1). Several of these genes, including MX, OAS, PKR, IFITM and IFIT5 expression have been implicated in influenza virus infection. MX gene is first reported ISGs that restrict influenza virus infection [44, 45]. IFITM proteins restrict the influenza A virus replication [46, 47]. The OAS family and RNase L act together to degrade viral RNA in the cytosol [48]. PKR protein restricts viral replication by inhibition of translation of viral mRNA [49, 50]. The IFIT family proteins recognize viral RNA with 5’triphosphate thereby IFIT complex antagonizes viruses by sequestering specific viral nucleic acids [51].

In spite of the fact that the host is induced to express a good number of IFN-stimulated genes and pro-inflammatory cytokines, influenza virus evade the host’s immune response and efficiently replicate in lung tissues. The influenza virus evade host’s immune response by induction of suppressor of cytokine signaling (SOCS), a potent endogenous inhibitor of TLR signaling and IFN signaling [52–56]. SOCS1 (fold change value 3.7), SOCS3 (fold change value 4.2), SOCS4 (fold change value 1.4), and SOCS5 (fold change value 2.3) genes were upregulated in HPAIV H5N1 infected lung tissues. Upregulation of SOCS genes inhibit the antiviral response of the host and pave the path for efficient virus replication in the lung tissues [27]. This increased viral load in the lung tissues induces excessive proinflammatory cytokines expressions [27]. Further activation of TNF signaling pathways induce increased expression levels of many pro-inflammatory cytokines [27]. TNF signaling pathway genes (CEBPB, CX3CL1, IL18R1, IL1B, MAP3K14, MMP14, PTGS2, SOCS3, TNFAIP3, TRAF2, TRAF3, VCAM1) and TNF super family genes (TNFAIP3, TNFAIP6, TNFRSF10B, TNFRSF13C, TNFRSF21, TNFSF13B, TNFSF15 and TNFSF8) were upregulated in HPAIV infected tissues.

Higher level expression of proinflammatory cytokines and chemokines, including IFNA, IFNG, TLRs, IL-1, IL-6, IL-12, IL-10, IL-18, TNF-α, CCL2, CCL4, CCL5, and CXCL10 were reported in humans and animal models infected with pandemic /H5N1 influenza viruses [10, 11,13–16]. Similarly the HPAI H5N1 infection resulted in very high transcriptional induction of IFNA [5, 57, 58], IFNG [5, 59], IL1B [5, 59], and IL6 [5, 57, 59, 60] was reported in chickens using RT-qPCR. IL-6 expression is directly linked to host morbidity [61, 62]. Chemokines expression at influenza virus infections correlates with disease severity and mortality [10, 15, 63]. This intense host inflammatory immune response cause permanent lung tissues damage result in critical organ failure and death of the host [5, 59, 60]. In summary, the present genome wide host gene expression study clearly indicates that the H5N1 virus induces hypercytokine responses resulted in high mortality in chickens.

**Fig 5:**
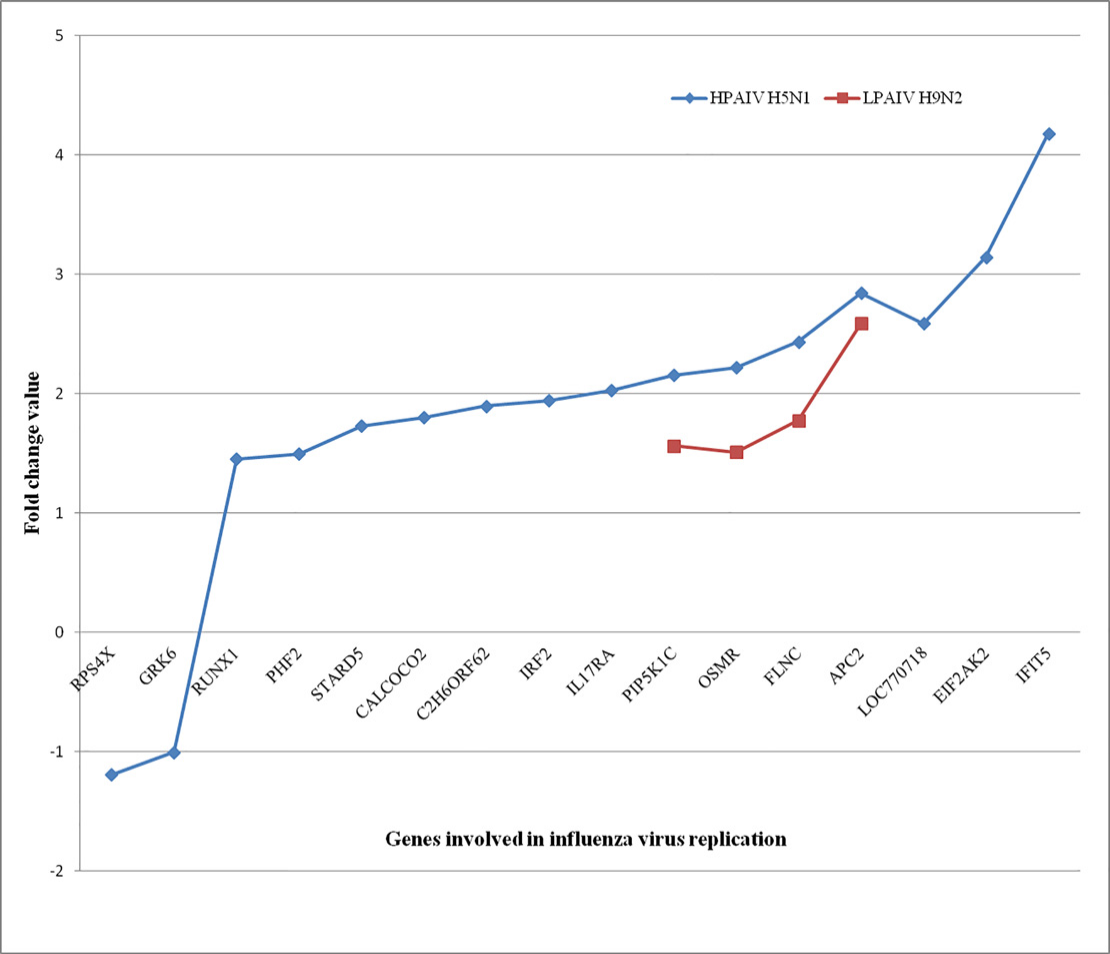
The host genes were involved in avian influenza virus replication in chicken lung tissues are shown along with fold change expression value.

Avian influenza virus replication in cells leads to the induction of apoptotic cell death and apoptosis has been suggested to contribute to the mortality of the host [2]. Activation of apoptosis pathways such as p53 signaling pathway, MAPK signaling pathway and PI3K-Akt signaling pathway were observed in HPAIV infected lung tissues (Fig 2). The expression of genes MAPK12, MAPK1IP1L, MAPK8IP3 and MAPKAP1 that are associated with cell death were also observed in H5N1 virus-infected tissues. Watanabe et al reported 128 human genes involved in influenza virus replication cycle [64]. Some of these genes such as RPS4X, GRK6, RUNX1, PHF2, STARD5, IRF2, IL17RA, APC2, and IFIT5 etc. were expressed in our transcriptome data (Fig 5).

### Immune response to LPAI H9N2 virus

Most low pathogenic avian influenza viruses cause no or mild disease in avian species. Little is known about immune response and molecular mechanism of tolerance to LPAIVs infection in birds [6]. To characterize the transcriptional immune response to LPAI H9N2 virus, we analyzed the differentially regulated genes in chicken lung tissues using microarray. Infection of chicken with LPAIV induces high levels of IFN in the lung tissues [28, 58, 65]. However in our case LPAIV H9N2 virus induced the expression of type I interferon (IFNA) with fold change value of 1.8 in lung tissues. Xing et al suggested that weak induction of IFNs in H9N2 infected macrophages may probably contributed to differential regulation of IFN-inducible genes [6]. Among the differentially regulated genes, IFITM5, TRIM50, and TGFB3 genes were downregulated in the lung tissues. Additionally, interleukins like IL12B, IL13, IL17F, IL23A, IL9A, and TGFA were all upregulated in LPAIV H9N2 infected lung tissues. The expression of other interleukins and chemokines genes were not induced in the lungs tissues of chickens infected with LPAI H9N2 virus.

The immune pathways activated in response to LPAIV infection in chickens were the TLR pathway, RIG-I pathway, Jak-STAT signaling pathway, NF-kappa B signaling pathway, Cytokine-cytokine receptor interaction, MAPK signaling pathway, TGF-beta signaling pathway, Chemokine signaling pathway etc. (Fig 3). Activation of these pathways in chicken lungs confirmed an innate immune response at the transcriptional level. Many of these immune pathways were previously reported to associate with influenza virus infection [2, 27, 56, 66, 67]. Maughan et al reported that ducks are able to tolerate negative consequences associated with LPAIV infection through fine-tune their innate immune response by differentially regulating the various immune related pathways [66]. Similarly, chickens may differently regulate these immune pathways to tolerate LPAIV infections.

In conclusion, highly pathogenic H5N1 virus induced excessive expression of innate immune genes in the lung tissues. This atypical expression of immune genes might be the cause for the high mortality in chickens. In contrast, the expression of most of these genes was either not induced or some were downregulated except for a modest expression of some immune genes such as IFNA, IFITM5, TGFB3, IL12B, IL13, IL17F were observed in LPAI H9N2 virus infection in chickens. Hence further studies are required to elucidate what host factors are controlling the gene expression during infection to recover or tolerate LPAIV infection in chickens.

## Supporting Information

S1 Fig Validation of microarray data by RT qPCR. Relative amount of differentially expressed genes in LPAIV H5N1 infected as compared to non infected lung tissues using RT qPCR.
(TIFF)
