# Supplementary figures and images for: Genome Wide Host Gene Expression Analysis in Chicken Lungs Infected with Avian Influenza Viruses
Source: PLoS One. 2016 Apr 12;11(4):e0153671. doi: 10.1371/journal.pone.0153671 (PMC4829244; doi:10.1371/journal.pone.0153671)

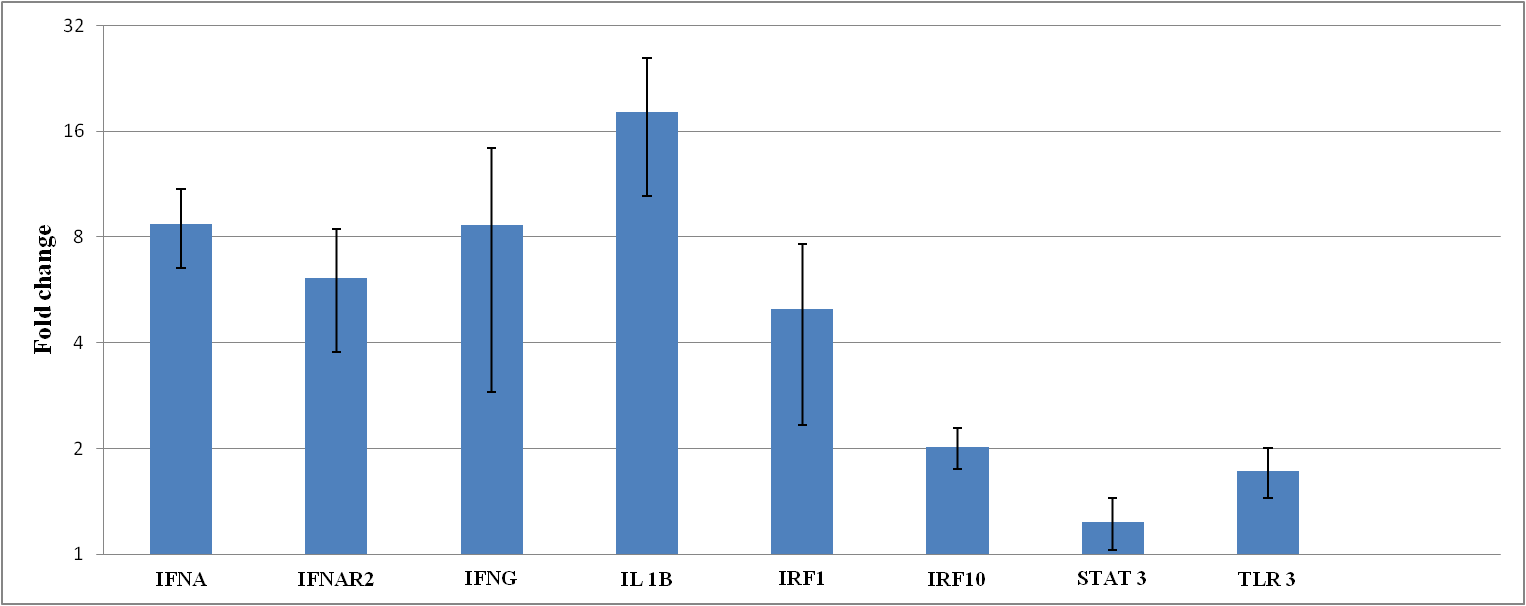

Supplement: S1 Fig — Relative amount of differentially expressed genes in LPAIV H5N1 infected as compared to non infected lung tissues using RT qPCR. (TIFF) [file pone.0153671.s001.tiff]
